# Death in the Church

**Published:** 1892-02-13

**Authors:** 


### The Hospital

A Weekly Journal of

Science, Medicine, Nursing, and Philanthropy.

*For Hospitals, Asylums, Infirmaries, Dispensaries, Medical Practitioners, Students, Nurses, and the Charitable Public.*

Vol. XI.—No. 281.] [Feb. 13, 1892.

### Death in the Church.

A favourite text with many of the younger clergy is " Death in the pot." Some correspondents in the *Standard* have made it evident that the risks are neither few nor small of " death in the church." The present writer has himself " suffered much at many churches "; and a fellow-feeling with the *Standard's* correspondents gives at least sincerity to his pen. A well-known parable incidentally illustrates the fact that " the children of this world are wiser in their generation than the children of light." If the general standard of " comfortableness " in churches represents the maximum of the sanitary wisdom of the children of light, it is quite certain that what was true of them in the first century of the Christian era is equally true in the nineteenth.

There are hundreds of persons killed in London every winter by bronchitis and inflammation of the lungs, who contract those fatal diseases whilst sitting in churches and chapels. This may be considered a bold statement to make. But it is not more bold than true. There are hundreds of clergymen and ministers who are the victims of chronic sore throat, bronchial catarrh, asthma, and cardiac irritability, who owe those distressing and life-shortening affections entirely to the insanitary condition of the buildings in which they conduct religious worship. Many persons make it a rule to abstain from attendance at church from the beginning of October to the end of March, except on those rare occasions when the weather happens to be both mild and dry.

Nobody need wonder at the hoarseness of the clergyman, the continual coughing of the congregation, and the general discomfort of the Sunday morning service in our town churches. We have a climate which in winter is the dampest of the damp, and more changeable even than a fickle woman. To manage the atmosphere which such a climate supplies us with inside a public building requires trained skill and unwearying attention. But what kind of person do we ordinarily employ to cleanse, warm, and ventilate our churches? Is it not the case that the sexton or church officer is very frequently a man who, having failed at half a score ordinary occupations, is foisted into his office by some sympathetic patron because every other resource has been exhausted except the parish? A man of this class would be just as likely to make a successful prime minister as a successful sexton. So far is it from being the case that the workman who has failed at every other occupation is likely to make a good enough sexton, that only the very best and most intelligent of workmen are in any sense fit for such an office.

It is worth while to try to put oneself in the place of an average sexton on such a damp and unwholesome morning as last Sunday was. The ordinary sexton is a man accustomed to going about in all sorts of weather without his coat, and with his shirt sleeves rolled up. The thing that struck him last Sunday was, not that the morning was damp, but that it was warm, or rather that it was not cold for the time of the year. He congratulated himself, no doubt, upon the mildness of the weather, because he thought it prevented the necessity of lighting the fire of the heating apparatus at an early hour, and getting up a comfortable temperature. Instead of rising early as usual, he lay an hour or two longer in bed, and contented himself with the easy task of lighting a little peep of gas at every burner half an hour before the time of the morning service. What was the result of this easy and, from the standpoint of the sexton's ignorance, entirely natural proceeding? This was the result, that when the congregation began to arrive they found the atmosphere of the church damp and chill as a charnel house. Every one of them who was already suffering from a cold immediately began to shiver, to cough, to breathe as if the chest were stuffed with wool, and to enter upon a relapse much more dangerous than the original cold. The more delicate amongst those who had not colds already, began to contract them within a quarter of an hour of their entrance into the church; and the service proved just sufficiently long to enable the initial stage of a naso-bronchial catarrh to complete itself. If our supposed church had a congregation of a thousand it is probable that at least fifty persons made existing colds worse, and that fifty others acquired new ones. That is decidedly a sharp penalty to pay for hearing a service read which might have been read at home, and for listening to a brief sermon which in all probability was of only a second or third rate order of merit.

Such were the things which our supposed sexton actually did. Now let us consider what he ought to have done. When he waked up at seven o'clock on Sunday morning, if he had been an intelligent man, he would have perceived that the air inside the church was a little too cold and a great deal too damp; he would have recognised also that it was stagnant air, and required to be moved about and renewed. He would have felt, too, with his fingers, that the wood of the pews, and the cushions and the books, and indeed everything in the church and in the vestry was damp and cold. He would thereupon have said to himself that the building needed to be warmed a very little, but dried thoroughly.

He would have lighted his fires by eight o'clock, and made the atmosphere inside quite hot. Then he would have opened all his windows and doors, and thus caused the stagnant air to be replaced by fresh and cold currents from the outside. This he would have heated in its turn until it was not only warm but largely freed from watery vapour. Then he would have lowered his fires, and brought down the temperature, until his conveniently placed thermometers stood at sixty degrees or a little more all over the church. He would have kept his fires at such a degree of heat as to have maintained this temperature until all the people were comfortably seated, and then he would have allowed them gradually to die down. The heat given off by the bodies of the congregation would have compensated for the want of heat from the dying fires. Everybody would have breathed dry air; nobody would have coughed; the congregation would have been cheerful; the clergymen would have read and preached with ease and freedom; and instead of bad colds having been made worse, and new ones contracted, the whole congregation, and the clergymen also, would have felt lighter, brighter, and better, even in bodily health, for the morning's worship.

One of the *Standard's* correspondents makes urgent complaint against the inefficient means of ventilation which are so grievously common in churches and chapels. He points his moral with the present influenza epidemic. There is no better use which could be made of the influenza than that of compelling attention to the extreme unwholesomeness and dangerousness of many of our churches and chapels. The prevalent epidemic, the correspondent is disposed to think, "is largely due to a vitiated atmosphere. Just as the Black Death, the Plague, the Gaol Fever, and the cholera were all of them scourges traceable to neglect of sanitary laws, so probably the present epidemic may also be attributed to the same cause. Microscopists are endeavouring to discover the influenza bacillus; it is more than probable that an analysis of the atmosphere of houses of assembly, of which places of worship are the principal, would bring home to us with certainty what is the real cause of the scourge."

The view that the influenza bacillus may be present and develope rapidly in vitiated atmospheric air is just as probable as that it develops with great energy in the fluids and passages of the human body. Be that as it may, the question of the sanitation and comfort of public buildings such as churches and chapels is one of the very first importance. Sunday is our day of rest and recreation. Religious worship is rest and recreation in the highest form. To make our churches and chapels not merely places of discomfort but of serious danger to health is to aim a deadly blow at religious worship and religious instruction themselves. To the average town dweller one day in seven of repose and refreshment of spirit is absolutely essential. But if a man discovers that his seat in church is draughty, or that the whole atmosphere of the building is poisonous and damp, his hour of spiritual refreshment is turned into an hour of nervous apprehension lest his stay should inflict upon him a cold in the chest which may keep him away from business for a whole week. The churches and chapels, and almost everything that is connected with them are carried on in the most elementary and happy-go-lucky kind of way. There is, perhaps, one exception to this somewhat sweeping rule. The collections, it must be admitted, are always punctually made, and their success is diligently prepared for. It is eminently discreditable to church managers that anybody writing in a public newspaper, and calling churches and chapels "death traps" should waken sympathising echoes in every part of the country. Let us hope that the present timely ventilation of the subject will lead to much-needed reforms.

